# Supplementary material for: Effect of interstitial palladium on plasmon-driven charge transfer in nanoparticle dimers
Source: Nat Commun. 2018 Apr 23;9:1608. doi: 10.1038/s41467-018-04066-2 (PMC5913128; doi:10.1038/s41467-018-04066-2)
Supplement: Supplementary file 1 — Supplementary Information [file 41467_2018_4066_MOESM1_ESM.pdf]

Supplementary Information for

Effect of Interstitial Palladium on Plasmon-Driven Charge Transfer in Nanoparticle Dimers

Sarah Lerch and Björn M. Reinhard*

*bmr@bu.edu

**Sample**	**Zeta Potential**
DNA/PEG dimers (in DI H_2O)	-24.0 mV (± mV)
DNA/PEG dimers, incubated with Pd^{2+} (in DI H_2O)	-8.0 mV (± mV)

Supplementary Table 1 Pd^{2+} ion binding changes the zeta potentials of the PR used in this work and indicates the binding of Pd^{2+} ions. Zeta potentials were measured after 4 hour incubation.

	**15 DNA/NP**	**22 DNA/NP**	**30 DNA/NP**
High Pd NP Loading	115	152	126
Percentage of Total	40.1%	49.8%	67.7%
Low Pd NP Loading	172	153	60
Percentage of Total	59.9%	50.2%	32.3%
Total Number of PRs	287	305	186

Supplementary Table 2 Pd NP were formed in PRs with different DNA/NP ratios (from left to right: 15/1, 22/1, 30/1) and inspected in the TEM. Based on the coverage of the PR with Pd NPs, the PRs were categorized as high (more than approx. 40 Pd NP) or low (less than approx. 40 Pd NP) loading.

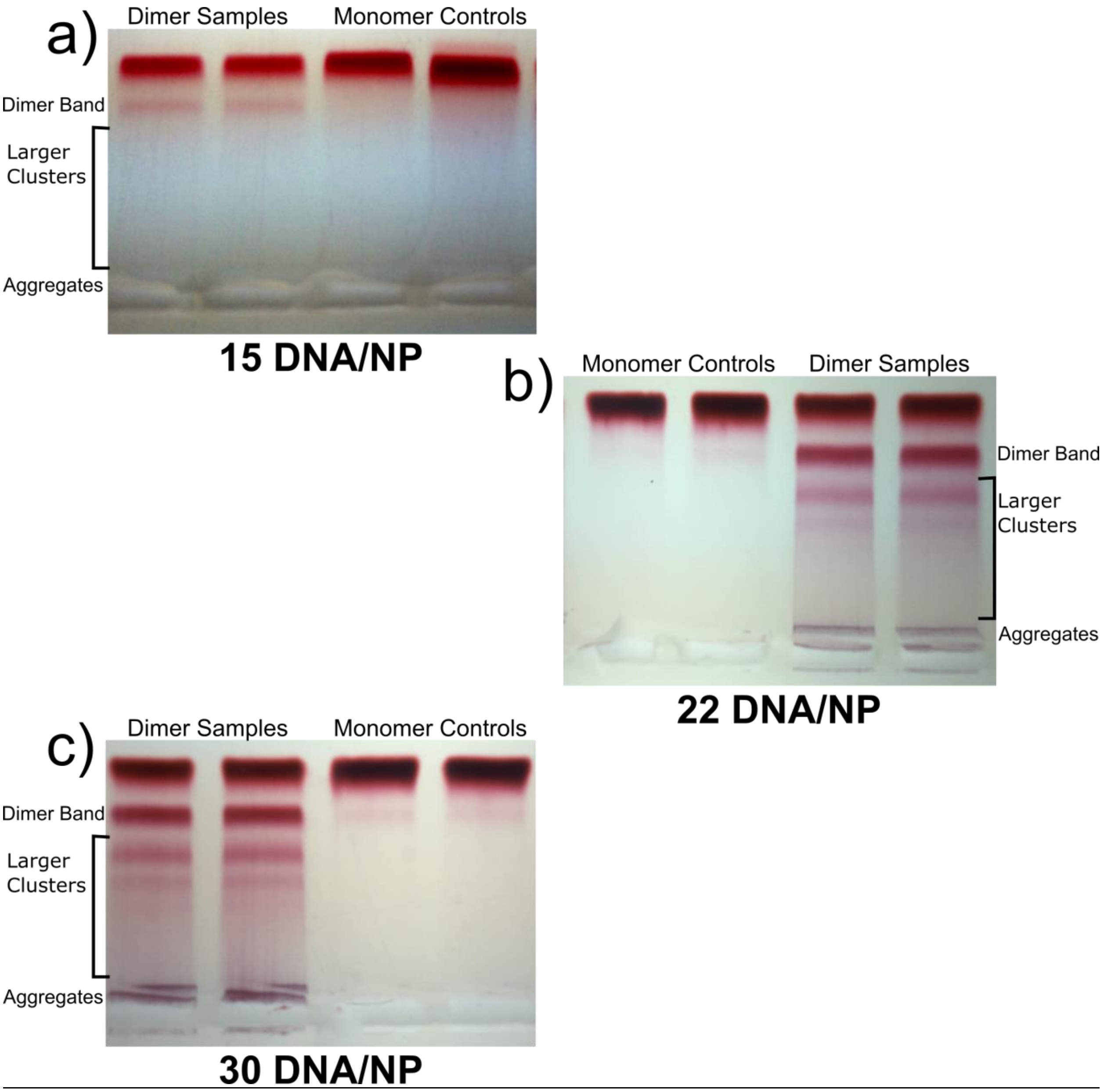

Supplementary Fig. 1 Images of gels used for the electrophoretic separation of plasmon rulers (PRs) with different DNA/NP ratios (15/1, 22/1, 30/1). Monomer controls are included for comparison. **(a)** The 15 DNA/NP sample shows a monomer band (77.5%) and a dimer band (22.5%) after 48 hours incubation at 60°C with no significant larger clusters and no aggregations. The %-values refer to the relative contributions of the individual species and were calculated from the integrated intensities of the individual bands. **(b)** 22 DNA/NP sample after 30 hours of incubation at 60°C. The gel shows a monomer band (45.5%), a strong dimer band (21.2%), a trimer band (11.8%) and faint tetramer band (9.5%, larger clusters), in addition to some aggregation (12%). The stronger contribution from larger assemblies when compared with (a) indicates a higher efficiency of hybridization due to increases DNA loading on the NPs. **(c)** 30 DNA/NP sample after 5 hours of incubation at 60°C shows a monomer band (42.7%), a strong dimer band (19.8%), a trimer band (11.7%), a tetramer band (10.1%), as well as significant

aggregation (15.7%). 30 DNA/NP samples are typically incubated at room temperature to avoid the formation of significant aggregations, but were incubated at 60°C here for consistency with (a) and (b).

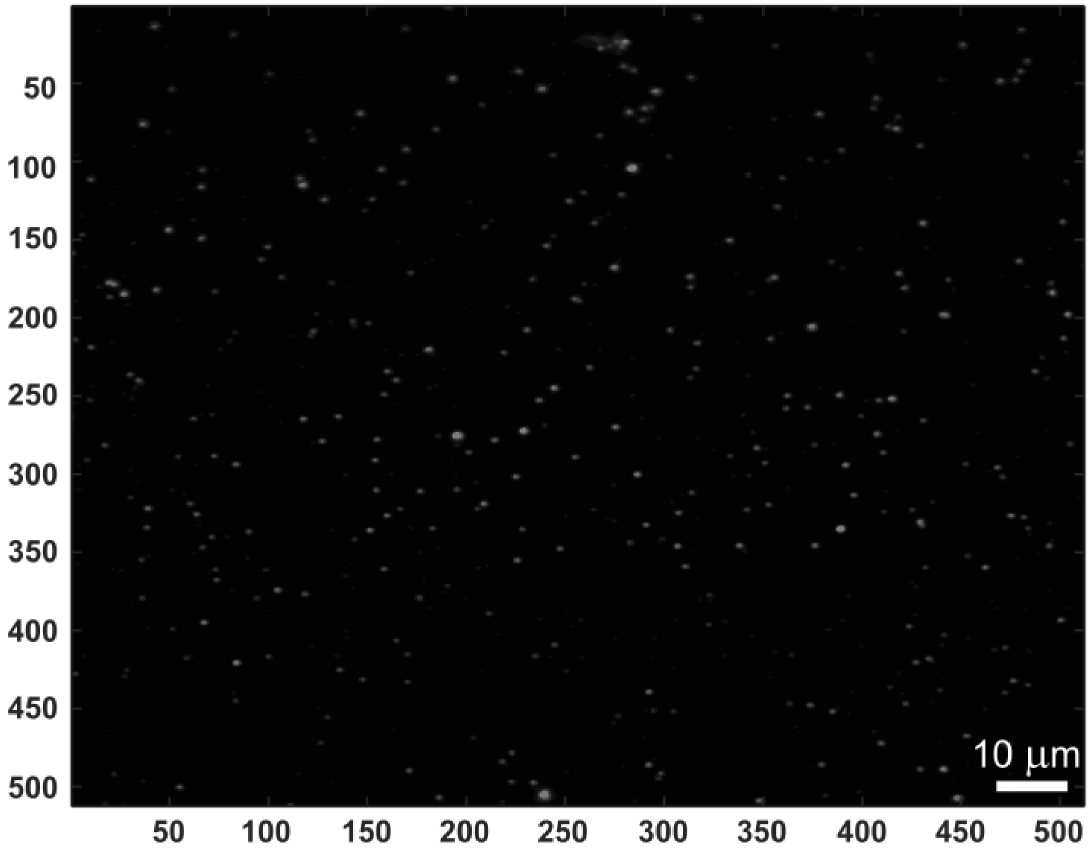

Supplementary Fig. 2 Monochromatic darkfield scattering image (full view of view) containing 595 discretely detectable scatterers. A smaller sub-field of this view is included in Figure 3a.

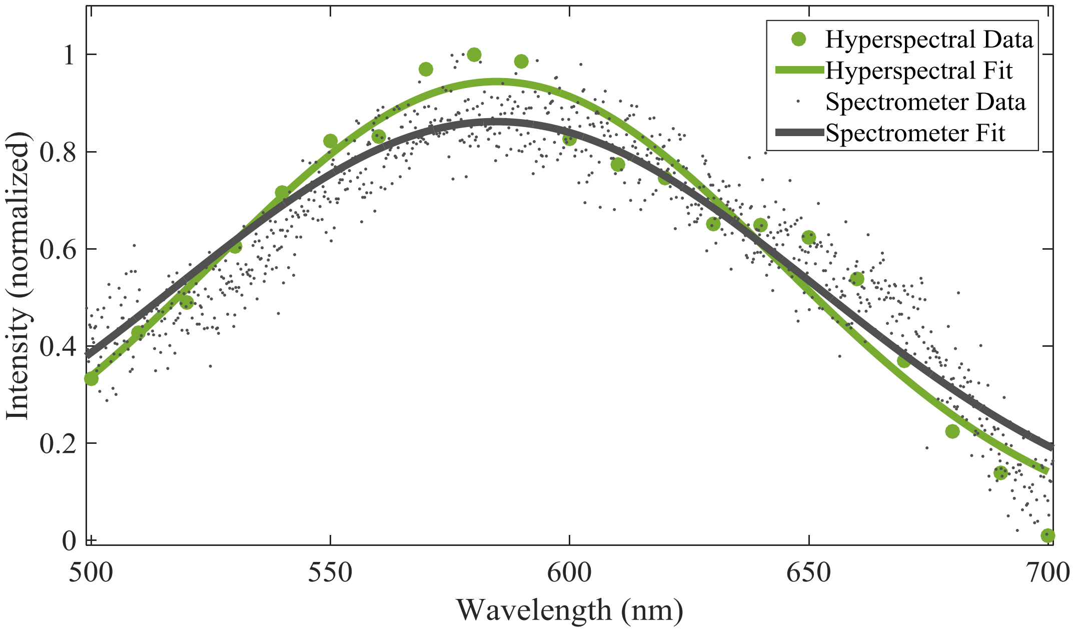

Supplementary Fig. 3 Example spectra showing accuracy of hyperspectral imaging technique (green) compared to a typical spectrometer (grey). Hyperspectral imaging techniques are detailed in the Methods section. Spectrometer data was collected with an Andor Shamrock spectrometer, centered at 600 nm.

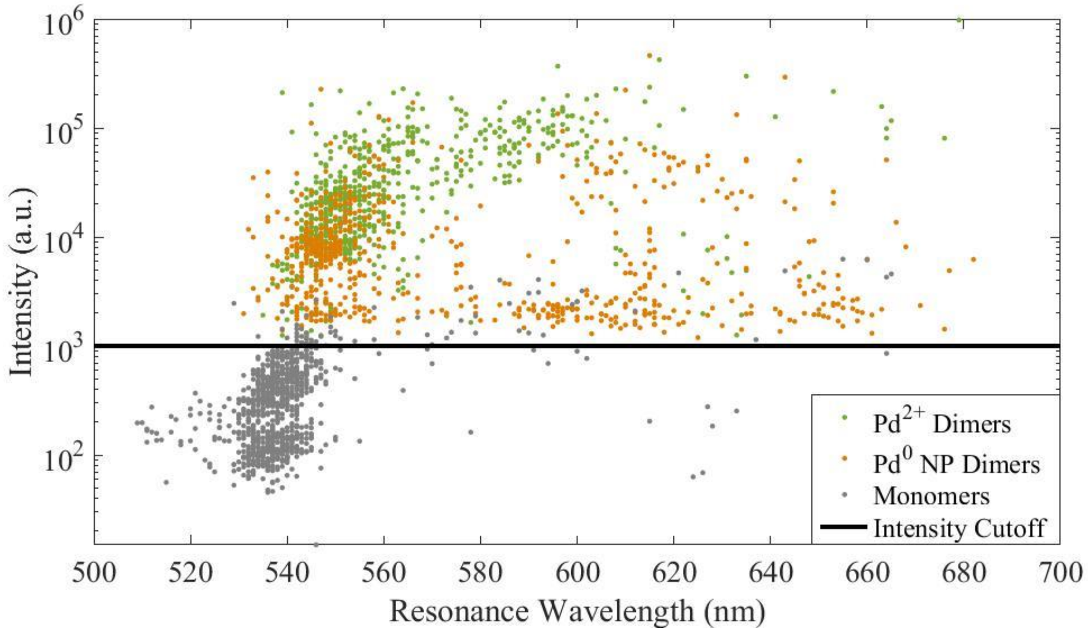

Supplementary Fig. 4 Intensity data for monomers (grey) and PRs before (green) & after (orange) reduction of Pd^{2+} to Pd NPs. Intensity cutoff to distinguish monomers from dimers is located at $1x10^3$ a.u. (black line) was used throughout the analyses in this paper. This intensity cutoff represents the average monomer intensity plus one standard deviation.

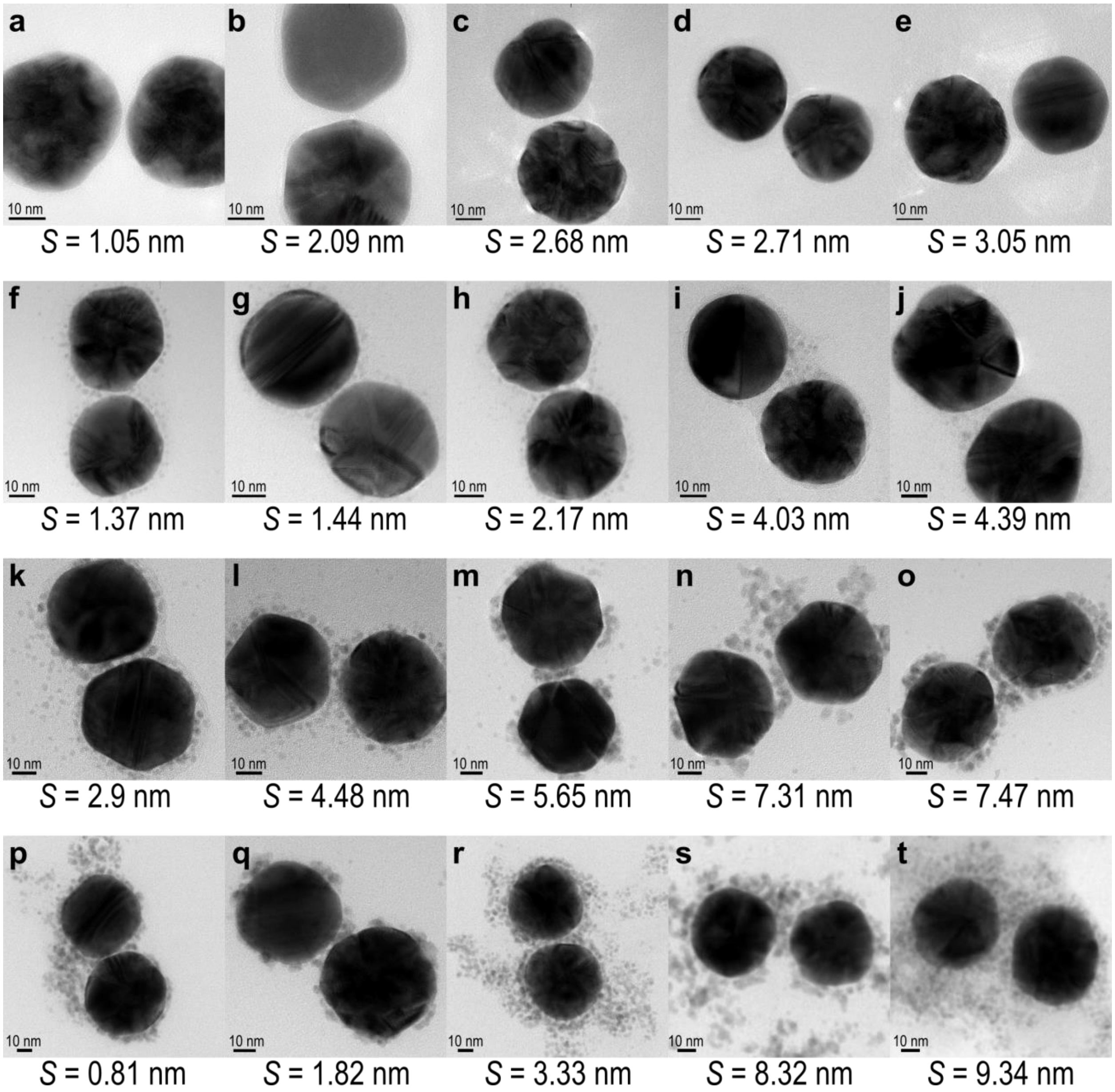

Supplementary Fig. 5 TEM images of PRs with only DNA (a-e), PRs containing discrete Pd NPs (f-j), PRs with Pd NPs forming a low number of point contacts between the Au NPs and/or partially filling the gap (k-o), and PRs containing an even higher density of Pd NPs so that the gap region is (nearly) completely filled and a broad region of contact between the Au NPs if formed through a dense Pd NP bridge whose end points touch the gold NPs (p-t). The spectra of these structures are summarized in Supplementary Fig. 6.

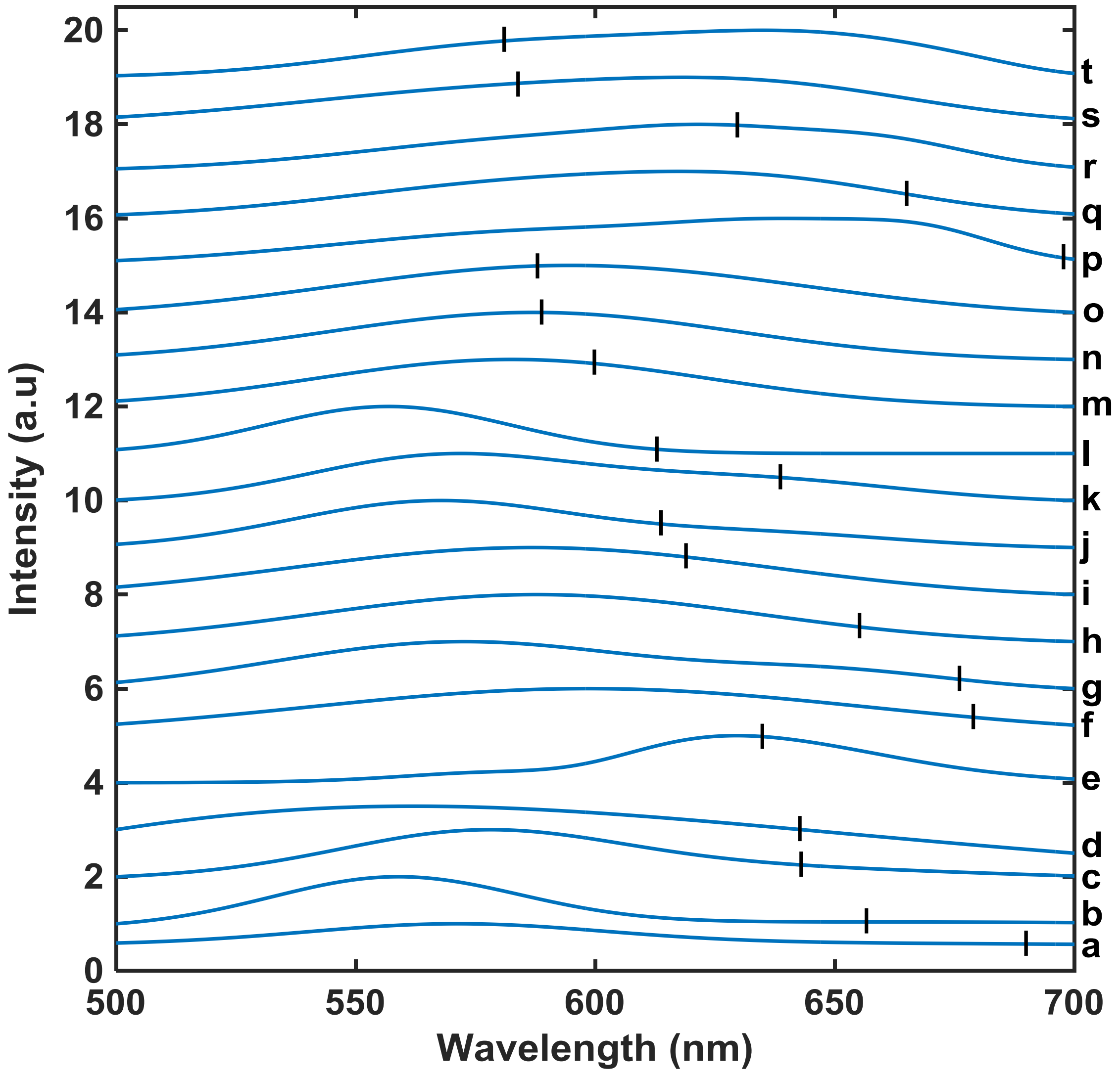

Supplementary Fig. 6 Additional spectra for PRs with DNA only or PRs containing Pd NPs, corresponding to numbered TEM images in Supplementary Fig. 5 above. Spectra are normalized and the expected peak resonance wavelength from FDTD simulations (black spectra in main text, Figure 4) is indicated by the black lines on each spectrum. Please note: PR j which shows a clear blue-shift between experimental spectrum and FDTD simulations has an interparticle separation of S = 4.39 nm.

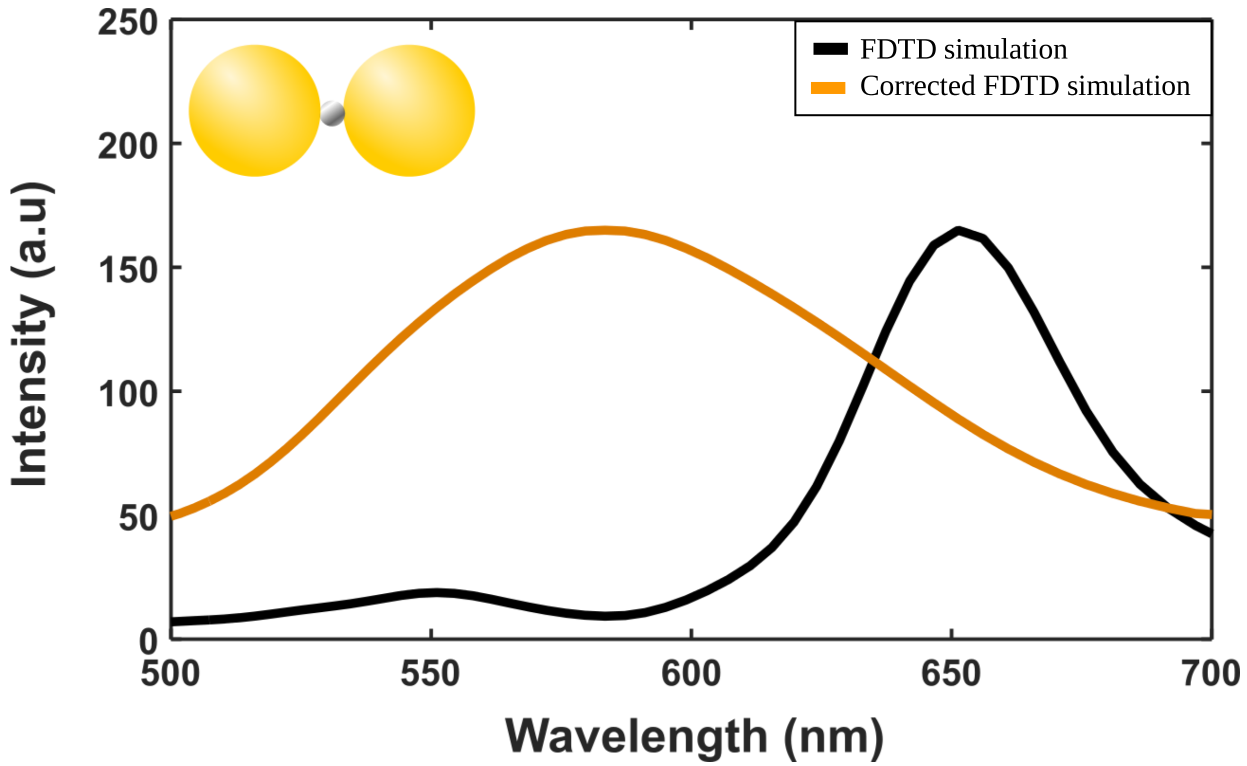

Supplementary Fig. 7 FDTD simulated spectra (orange) for a PR (S = 2 nm, λ_{corr} = 581 nm) conductively bridged by a 2 nm Pd NP (see text). This structure experiences a similar shift for the PR modelled in Fig. 4a, due to an increase in the gap conductance when compared to the same PR in vacuum (black, λ_{sim} = 651 nm).

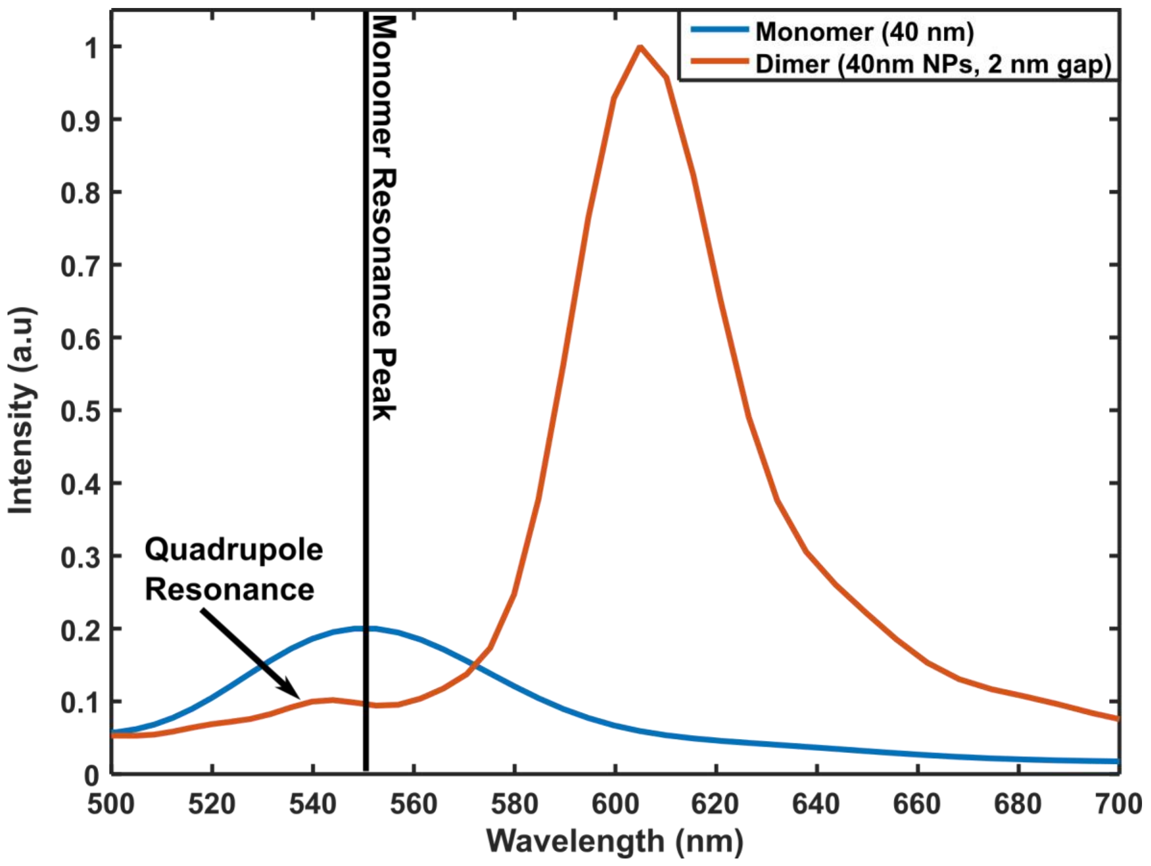

Supplementary Fig. 8 FDTD simulated spectra for a 40nm Au NP monomer (blue) and a 40nm Au NP dimer with a 2 nm gap (orange) showing the quadrupolar mode lies blueshifted to the monomer resonance. This rules out multipolar radiation for the blue-shifted resonance that remains in the main text Figure 4d, as that mode is not shifted further than the monomer resonance. Additional confirmation can be found in the work by Atay, et al.[1]

Supplementary References

1. Atay, T., Song, J.-H. & Nurmikko, A. V. Strongly Interacting Plasmon Nanoparticle Pairs: From Dipole−Dipole Interaction to Conductively Coupled Regime. *Nano Lett.* **4,** 1627–1631 (2004).
